# 1-day bowel preparation with polyethylene glycol 3350 is as effective and safe as a 3-day preparation for colonoscopy in children

**DOI:** 10.1186/1756-0500-7-648

**Published:** 2014-09-15

**Authors:** Serge A Sorser, Venkata Konanki, Alice Hursh, Karen Hagglund, Hernando Lyons

**Affiliations:** Department of Gastroenterology, Providence Hospital and Medical Center, Southfield, MI USA; Department of Pediatrics, Chambersburg Hospital, Chambersburg, PA USA; Department of Pediatric Gastroenterology, British Columbia Children’s Hospital, Vancouver, British Columbia Canada; Department of Medical Education, St. John Hospital and Medical Center, Detroit, MI USA; Department of Pediatric Gastroenterology, St. John Hospital and Medical Center, Wayne State University School of Medicine, Detroit, MI USA

**Keywords:** Pediatric colonoscopy, Bowel preparation, Safety, Tolerability

## Abstract

**Background:**

Polyethylene Glycol 3350 (Miralax®) without electrolytes is commonly used for 3–4 days as bowel preparation for colonoscopy in children. One-day preparation has been anecdotally reported to be effective but there are few published prospective studies comparing the safety and efficacy of one-day preparation with that of three-day preparation. This study was conducted to compare the efficacy and safety of a one-day bowel preparation with Miralax® with that of a three-day preparation for colonoscopy in children.

**Methods:**

We conducted a prospective, randomized controlled trial with children age 2–21 yrs. undergoing elective colonoscopy. Patients were randomly assigned to receive Miralax® for either one or three days. Children with known electrolyte disturbances, dehydration, fecal impaction, metabolic or renal disease were excluded. A metabolic panel was monitored before and after bowel preparation. Subjects reported the tolerability and side effects of Miralax® via a survey. Effectiveness of the bowel preparation was assessed using a stool diary and a bowel cleansing scale during colonoscopy.

**Results:**

32 subjects were enrolled; 18 received one-day bowel preparation and 14 received 3-day preparation. There were no differences between the groups in efficacy of bowel preparation based on colonoscopic grading or the safety of the preparation. One-day preparation was as well tolerated as three-day preparation.

**Conclusion:**

Miralax® used one day as bowel preparation for elective colonoscopy in children is safe, effective and well tolerated. Physicians should consider offering a one-day option for bowel preparation, which would allow children to miss fewer days of school prior to colonoscopy.

**Trial registration:**

Trial Registration Number: NCT02174497. Date of Registration: 02 May, 2014 URL of register: clinicaltrials.gov.

## Background

Colonoscopy in children and young adults is performed by gastroenterologists for a variety of indications, including but not limited to evaluation of rectal bleeding, chronic diarrhea, family history of polyposis syndromes and surveillance for colon cancer in patients with inflammatory bowel disease [1–4]. A successful bowel preparation that enables clear visualization of the intestinal mucosa is essential to diagnose and treat the underlying pathology. Currently, gastroenterologists use a variety of colon cleansing preparations, but Polyethylene Glycol (PEG) 3350 given for three to four days appears to be standard [5–8]. Though it is well tolerated, safe and effective [5, 9–11], the duration of preparation may cause disruption to the routine and missed school days. To date, there is limited data that shows efficacy of a shorter duration of bowel preparation with PEG [12–14]. This study aims to compare the efficacy and safety of a one-day preparation with that of a three-day preparation with PEG 3350 as preparation for colonoscopy in children and young adults.

## Methods

A prospective, randomized controlled study was conducted at St. John Hospital and Medical Center in Detroit, MI between February 2008 and February 2010, after obtaining Institutional Review Board (IRB) approval from the St. John Hospital IRB, which provides oversight for both the scientific and ethical aspects of research studies conducted at our facility. Children between 2 and 21 years of age undergoing elective colonoscopy were enrolled into the study. The exclusion criteria for this study were a history of allergy to PEG, fecal impaction, known electrolyte abnormalities, as well as renal and metabolic diseases. Written consent from subjects or their parents was obtained at the time of enrollment.

Subjects were randomly assigned to either a one-day or a three-day preparation group. Those in the one-day group were instructed to take 4.5gm/kg/day PEG 3350 up to a maximum dose of 255 g and those in the three-day group to receive 1.5 g/kg/day, up to a daily maximum dose of 85gm and total dose of 255 g. Written instructions were provided to take PEG with any clear liquid. Subjects were asked to take 1 scoop (17 g) of PEG mixed with 8 oz. of clear liquid every 30 minutes. Those under 7 years of age were asked to take ½ scoop (8.5 g) mixed with 4 oz. of clear liquid every 30 minutes. Subjects in the three-day group were instructed to take a regular diet for the first two days of preparation and a clear liquid diet on the 3^rd^ day. Subjects in the one-day group were instructed to take clear liquid diet on the day of bowel preparation. Subjects in both groups were asked not to take anything by mouth (NPO) eight hours before procedure except sips of water up to three hours before procedure.

Demographic data such as age, gender, race, ethnicity and weight were collected. Indications for colonoscopy were recorded. Subjects were given written instructions regarding the total amount of PEG to be taken over one-day or three-days, what to take it with, in terms of quantity and frequency, and diet while undergoing bowel preparation.

Subjects were asked to report how tolerable (easy, tolerable or difficult) the preparation was and whether they completely took PEG as prescribed. If the patient did not take the entire prescribed regimen, reasons for not completing the preparation were elucidated. Subjects were also asked if they were willing to take the preparation again in the future, if they had to. Serum electrolytes, blood urea nitrogen (BUN), creatinine and serum osmolality were checked before and after the bowel preparation to monitor the safety of the preparation. While undergoing the preparation, a stool diary consisting of stool frequency, color and consistency was completed by all subjects.

At the time of colonoscopy, the pediatric gastroenterologist (HL) involved in the study, who was blinded to the preparation given, graded the degree of bowel cleansing as poor, fair, good or excellent depending on the type and amount of liquid debris or stool present. Endoscopic images were also evaluated by a blind reviewer (a second pediatric gastroenterologist, MEB). All segments of the colon were evaluated (cecum, ascending colon, transverse colon, descending colon, sigmoid colon, rectum), with photographs of each segment reviewed independently by the primary endoscopist and the blinded reviewer. A previously described grading system was used for this study [5]. A grading of excellent required no stool or small amount of thin fecal fluid to be seen. A good grading was characterized by liquid fecal matter that could be removed by suction easily. A grading of fair meant that thick stool was present, requiring suction with water lavage. A grading of poor required solid stools present with poor visualization of the mucosa. A grading of good or excellent was acceptable for the evaluation of the colonic mucosa and a repeat colonoscopy was not required. Primary outcomes measured were efficacy, safety and tolerability of PEG given one-day vs. three days for bowel preparation for colonoscopy.

Frequency and descriptive statistics were calculated. Associations between categorical variables and treatment group were measured by Chi-square or Fisher’s exact tests. Differences between groups on continuous variables were assessed using Student’s t-tests. P values < 0.05 were considered to indicate statistical significance. SPSS version 12.0 was used for statistical analysis.

Funding for this study was provided by the St. John Hospital & Medical Center Graduate Medical Education Research Committee.

## Results

A total of 32 subjects, 18 in the one-day group and 14 in three-day group, were recruited and included in the data analysis. No patients were excluded from this study based on the exclusion criteria listed. The mean age of subjects was 13.6 and 11.6 years in the one and three day groups, respectively. In the one-day prep group, 8/18 subjects were male, whereas, 10/14 subjects were males in the three-day group. The majority of patients in both groups, 15/18 in the one-day group and 12/14 in the three-day group, were Caucasian. In both groups, the most common indication for colonoscopy was rectal bleeding (11/18 in the one-day group versus 10/14 in the three-day group). Other indications were chronic diarrhea, abdominal pain, family history of familial polyposis syndrome and Crohn’s disease. This data is summarized in Table 1.

**Table 1:** **Demographics and indications for colonoscopy**

Demographics	1-Day prep	3-Day prep	P
	n = 18	n = 14	
	Mean ± SD or n (%)	Mean ± SD or n (%)	
Age	13.6 + 4.6	11.6 + 4.9	0.237
Gender			0.165
Male	8 (44)	10 (71)	
Female	10 (56)	4 (29)	
Race			1
Caucasian	15 (83)	12 (86)	
African-American	3 (17)	2 (14)	
**Indication for colonoscopy**			
Rectal bleeding	11 (61)	10 (71)	1
Chronic diarrhea	6 (33)	4 (29)	0.712
Abdominal pain	4 (22)	8 (57)	0.068
Crohn’s disease	2 (11)	0 (0)	0.492

The vast majority of patients, 16 in the one-day group and all 14 in the three-day group, indicated that the bowel preparation was either acceptable or tolerable, but this was not statistically significant. Most patients, 13/18 in the one-day group and 13/14 in the three-day group, took the full prescribed amount of PEG. The remaining patients, 5/18 in the one-day group and 1/14 in the three-day group took partial amount of PEG because they indicated that their stools were clear and did not take the remaining PEG. Almost all of the patients, 16/18 in the one-day and 14/14 in the three-day group indicated that they were willing to take their respective preparations again, if needed. Again, these were not statistically significant (p = 0.492). Tolerability data is listed in Table 2.

**Table 2:** **Tolerability of PEG**

	1-Day prep	3-Day prep	P
	n = 18	n = 14	
	n (%)	n (%)	
**Ease of administration**			0.492
Easy or tolerable	16 (89)	14 (100)	
Difficult	2 (11)	0 (0)	
**Compliance**			0.196
Full	13 (72)	13 (93)	
Partial	5 (28)	1 (7)	
**Willingness to take again**			0.492
Yes	16 (89)	14 (100)	
No	2 (11)	0 (0)	

Based on a stool diary completed by the subjects or their parents, the bowel preparation was assessed to be excellent in 16/18 in the one-day group and in 12/14 in the three-day group. Based on the endoscopic evaluation of bowel cleansing, a grading of excellent or good was given to 18/18 (14/18 excellent and 4/18 good) in the one-day group and 13/14 (9/14 excellent and 4/14 good) in the three-day group. The remaining patient in the three-day group received a grading of fair and that patient required a repeat endoscopic examination. The blinded reviewer gave a grading of good or excellent for all patients in both groups (9/18 excellent and 9/18 good for the one-day group and 7/14 excellent and 7/14 good for the three-day group). Agreement was reached in 22/32 patients (68.8%) The overall kappa correlation for the 32 cases between the two reviewers was 0.375, with a kappa of 0.571 for the three-day group and 0.222 for the one-day group. Please refer to Table 3 for data on efficacy of PEG in the one and three day groups.

**Table 3:** **Efficacy of PEG**

	1-Day preps	3-Day prep	P
	n = 18	n = 14	
	n (%)	n (%)	
**Effectiveness of bowel prep**			1
Excellent	16 (89)	12 (85)	
Good	2 (11)	2 (15)	
**Colonoscopic evaluation of prep**			0.437
Excellent or good	18 (100)	13 (93)	
Fair	0 (0)	1 (7)	
**Repeat colonoscopy needed**	0 (0)	1 (7)	0.452

The major side effects reported by patients were nausea, vomiting and abdominal pain. In the one-day group, 8/18 complained of nausea, 1/18 noted vomiting and 4/18 described abdominal pain, whereas 3/14 complained of nausea, 2/14 of vomiting and 3/14 of abdominal pain in three-day group. These symptoms were mild to moderate in both groups. The incidence of these side effects was not statistically significantly different between the two groups. All adverse events are summarized in Table 4.

**Table 4:** **Safety of PEG**

	1-Day prep	3-Day prep	P
	n = 18	n = 14	
	n (%)	n (%)	
**Side effects**			
Nausea	8 (44)	3 (22)	0.266
Vomiting	1 (6)	2 (14)	0.568
Abdominal pain	4 (22)	3 (21)	1
**Labs before the prep**			1
Normal	16 (89)	12 (86)	
Abnormal	2 (11)	2 (14)	
**Labs after the prep**			1
Normal	13 (77)	10 (71)	
Abnormal	5 (23)	4 (29)	

Review of serologic evaluation showed 2/18 in the one-day group and 2/14 in the three-day group had slightly low osmolality (277 milliosmoles/kg and 278 millosmoles/kg in the one-day group and 274 milliosmoles/kg and 275 millosmoles/kg in the three-day group) on pre-bowel preparation labs performed as part of the study. These were not associated with any clinical symptoms. Similarly, 5/18 in the one-day and 4/14 in the three-day group had abnormal serology following the preparation. In the one-day group, one patient had an elevated osmolality of 315, two patients had a low osmolality of 275 & 277 and two patients had low serum bicarbonate of 19 & 20. In the three-day group, two patients had low osmolality of 275 & 277 and two had low bicarbonate of 15 & 17. These serologic derangements were not associated with any clinical symptoms and the incidence of these abnormalities was not statistically significantly different between the two groups.

## Discussion

The importance of a safe, easily tolerable and effective colonoscopy preparation, particularly in the pediatric population, is paramount for both diagnostic and, at times, therapeutic reasons. This is important for the patient in terms of acceptability and missed days of school and his/her parents, in terms of taking time off of work. This is one of the first prospective studies evaluating the use of PEG 3350 without electrolytes for a one-day bowel preparation for colonoscopy in children and young adults in comparison with the three-day preparation. The data from our study indicates that the safety, efficacy and tolerability of bowel preparation with PEG given one-day are not inferior to that of three-day preparation.

At our institution, PEG is usually used as a three day bowel preparation in children. A recent retrospective study by Adamiak et al. reported an efficacy of 93% with one-day use of PEG for bowel preparation in children [12]. In our patient population, a similar efficacy (100%) with one-day preparation was noted.

The side effects reported in both groups in this study have previously been reported with use of PEG for bowel preparation as well as for other indications such as constipation, fecal impaction and bowel irrigation. Although there was a slightly higher incidence of side effects in the one-day group compared to three-day group, they were well tolerated and were not statistically significant. Following completion of the bowel preparation, low serum bicarbonate and low osmolality were noted in two patients in both groups, but they were of no clinical significance in this study. As the patients were asymptomatic, repeat serologic evaluation was not undertaken at a later time to document normalization.

In the one-day group, 89% of patients reported that the preparation was easy or tolerable and 72% completely took the prescribed amount of PEG based on their weight. Those that ingested only part of the preparation reported that their stools were clear and they did not feel that it was necessary to take the remaining PEG. This did not affect the quality of bowel cleansing.

The small sample size is an important limitation of our study. This was due to a slow recruitment rate at our institution. Another possible limitation of this study is evaluation bias by the gastroenterologist evaluating the degree of bowel cleaning at colonoscopy. However, this has been eliminated by having an independent gastroenterologist (blind reviewer), not involved in the study, review the endoscopic images and grade the preparation.

## Conclusion

The data from our study suggests that bowel preparation with PEG given as a one-day preparation is tolerable, safe and effective, and is not inferior to a three-day preparation. A shorter duration of bowel preparation will increase compliance, reduce the number of school days missed for children and workdays missed for their parents. However, future studies with a larger sample size are necessary to confirm these results. Furthermore, consideration for split dose preparation, as is becoming the standard in the adult population undergoing colonoscopy, may be considered [15–17] as a part of future studies.
